# Social influence aggregation on Weibo: psychological process expectations and meso-level cascade regimes in media psychology

**DOI:** 10.3389/fpsyg.2026.1953616

**Published:** 2026-10-21

**Authors:** Lulu Wang

**Affiliations:** School of Arts, Northeastern University, Shenyang, China

**Keywords:** digital gatekeeping, information cascade, media psychology, social contagion, social influence, social proof, uncertainty reduction, Weibo

## Abstract

**Background:**

Media Psychology investigates how media environments organize attention, judgment, and social influence. On Weibo, the repost is a publicly observable sharing act through which users may reduce uncertainty, follow social proof, and respond to intermediary visibility. A persistent research problem is that studies often move from laboratory exposures or message features to virality outcomes without specifying how micro forwarding decisions aggregate into distinct meso-level influence ecologies.

**Objectives:**

This article analyzes Weibo repost cascades as observational records of social-influence aggregation and asks whether deep–narrow (DN) vs. shallow–broad (SB) topology regimes differ in structural indicators that map onto chain-relayed influence, local-reinforcement opportunity, and brokerage.

**Methods:**

Using the public Key Propagation Graph Generator (KPG)/The Chinese University of Hong Kong (CUHK) Weibo propagation corpus (November 2019–March 2022; ≈4,174 source events), rooted trees were reconstructed from parent–child graph exports that also include term frequency–inverse document frequency (TF–IDF) feature vectors (text itself is unused here). Eligible cascades with 20–250 nodes (*N*_eligible_ = 1, 013) were partitioned by *k*-means (*k* = 2; silhouette = 0.513) on standardized depth and relative breadth; before balanced sampling, the pools contained 399 deep–narrow (DN) and 614 SB events. Size-tertile stratified draws yielded 40 DN and 40 SB cascades (*N* = 80; 6,527 nodes; 6,447 edges). Analyses used Python 3.12 with networkx (graph metrics), scipy (Welch tests), pandas, and matplotlib. Confirmatory DN/SB contrasts used Welch *t*-tests with Holm–Bonferroni adjustment; average path length was also regressed on continuous depth and breadth by ordinary least-squares (OLS).

**Results:**

Supporting confirmatory H1–H2, DN cascades showed longer average path lengths (Cohen's *d* = 2.71) and higher modularity (*d* = 2.95) than SB cascades. Density and clustering did not separate regimes after Holm adjustment. Exploratory role-proxy betweenness was descriptively elevated under DN for high-, medium-, and low-branching nodes; hop-layer branching persisted deeper under DN. These effect sizes quantify a regime-level separation of influence opportunity structures rather than merely restating depth.

**Conclusion:**

Social influence on Weibo aggregates into at least two structurally distinct ecologies—chain-relayed reinforcement opportunity vs. hub-broadcast exposure. The study supplies meso indicators for Media Psychology; it does not measure individual affect, attitudes, or uncertainty reduction as intrapersonal processes, and observational associations should not be read as causal psychological mechanisms.

## 1 Introduction

Media Psychology asks how media environments shape attention, judgment, emotion, and social influence in everyday life (Allport and Postman, 1947; Lazer et al., 2018). The present article is written for the Media Psychology specialty because its object is not graph topology for its own sake, but the psychology of online social influence as revealed in publicly observable sharing behavior. On Weibo, the elemental behavior is the *repost*: a decision to relay content under uncertainty, social proof, and intermediary visibility (Allport and Postman, 1947; Centola, 2010; Chadwick, 2013). When many such decisions aggregate, they form information cascades. Cascades are therefore behavioral traces of social influence—comparable in spirit to observational records used elsewhere in social and Media Psychology—rather than purely technical diffusion objects. The media-psychological question is how those micro decisions leave meso-level footprints that distinguish different *influence ecologies*.

Existing Frontiers research has already connected psychological constructs to social-media structure from multiple angles. Inferred personality traits have been linked to echo-chamber homophily on Weibo and Twitter (Song et al., 2024); experimentally induced information overload on Weibo has been shown to change attitudes (Wang et al., 2021); collective anxiety has been examined in topic-based online communities (Yang et al., 2021); coded emotional valence and arousal have been shown to predict retweet probability (Pivecka et al., 2022); and seeding strategies have been studied under complex-network structure (Zheng et al., 2022). Complementary work on expression capacity and negative-sentiment cascades further shows that influencer identification and structural monitoring can be integrated into mitigation designs (Rasheed et al., 2025; Ashraf, 2026). Across these designs, a shared insight is that psychological processes leave patterned traces in interaction structure.

Despite this progress, several limitations remain for cascade-focused Media Psychology.

First, studies often move from content labels (e.g., rumor/non-rumor) or laboratory exposures straight to virality outcomes, without specifying how forwarding decisions aggregate into cascade *regimes*—elongated multi-hop chains vs. early hub broadcasts (Romero et al., 2011; Weng et al., 2013; Vosoughi et al., 2018). Media Psychology therefore still lacks a clear account of when sharing produces chain-relayed influence contexts and when it produces broadcast-exposure contexts.

Second, although prior work has examined psychological experiences and predictors of sharing—including attitude change, emotion, collective anxiety, and personality-related homophily (Wang et al., 2021; Pivecka et al., 2022; Yang et al., 2021; Song et al., 2024)—systematic comparison of confirmatory *influence-aggregation indicators* across cascade regimes remains limited. Knowing that influence occurred is not enough; Media Psychology also needs indicators of *how* influence was socially organized.

Third, multi-level media-psychology designs risk jumping from individual measures (attitude, emotion, personality) to macro outcomes (virality, polarization) without a meso construct that describes aggregation. Regime-level differences can supply that missing middle layer for subsequent process measurement.

From a psychological perspective, users' forwarding decisions often arise from situational appraisal under uncertainty, social proof, and perceived influence of intermediaries (Allport and Postman, 1947; Centola, 2010). Different cascade geometries correspond, at least potentially, to different influence contexts: sequential peer reinforcement along chains vs. rapid hub exposure in early broadcast windows (Kwak et al., 2010; Romero et al., 2011). On this basis, cascade regimes can be treated as meso signatures of influence aggregation. Importantly, cascade shape must not be equated with content veracity, persuasiveness, or emotional intensity (Vosoughi et al., 2018; Zhou and Zafarani, 2021). Shape is a structural outcome of aggregated forwarding; interpreting it as “more emotional” or “more harmful” without micro measures is a category error that Media Psychology should avoid.

Accordingly, authentic Weibo repost trees are analyzed here as observational records of social-influence aggregation. The study asks how cascade regimes differ in indicators that map onto chain-relayed influence, local reinforcement, and brokerage; specifically, the following research questions (RQs) and hypotheses:

- **RQ1:** Do deep–narrow (DN) and shallow–broad (SB) Weibo cascade regimes differ in confirmatory indicators of social-influence aggregation (especially path length and modularity)?
- **RQ2:** Do hop-layer branching patterns show continued influence opportunity under DN relative to SB?
- **RQ3:** Is structural brokerage (betweenness) concentrated differently across high-/medium-/low-branching role proxies under DN vs. SB?

- **H1 (chain-relayed social influence):** DN cascades exhibit longer average path lengths than SB cascades, indexing more sequential influence opportunities.
- **H2 (local reinforcement clusters):** DN cascades exhibit higher modularity than SB cascades, consistent with fragmented clusters in which local social proof can accumulate (Centola, 2010; Yang et al., 2021; Song et al., 2024).
- **H3 (exploratory; brokered attention control):** mean betweenness of high-, medium-, and low-branching role proxies is descriptively higher under DN than under SB (Burt, 2004; Chadwick, 2013; Zheng et al., 2022). H3 is *not* included in the Holm-adjusted confirmatory family.

Methodologically, rooted cascades are reconstructed, DN/SB regimes are derived by unsupervised shape clustering, and confirmatory indicators are tested with multiplicity control. Secondary hop-layer and betweenness analyses elaborate process and gatekeeping interpretations. Full microblog text is unavailable; therefore, the study does not claim emotion coding or topic modeling (Pivecka et al., 2022). This is a deliberate Media Psychology scope choice: aggregation of sharing behavior is studied first, then how future work can attach self-reports and coded affect is specified. Figure 1 summarizes the psychological–structural bridge model.

**Figure 1:**
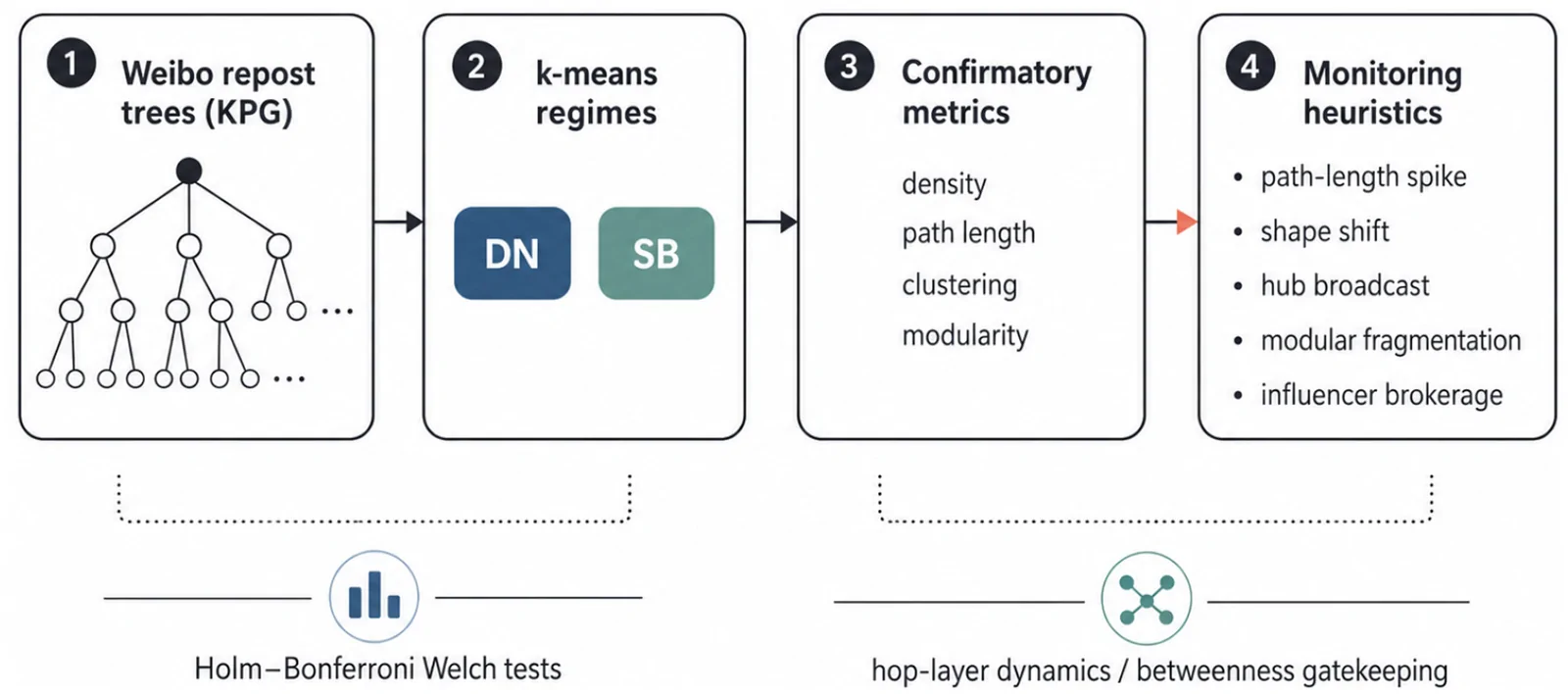
Psychological–structural bridge model used in this study. Micro process expectations motivate forwarding; meso cascade regimes (DN vs. SB) summarize aggregation (empirical focus); macro gatekeeping implications concern brokerage and modular fragmentation. Emotion coding and individual self-reports are reserved for future multi-level extensions.

The contributions of this study are threefold for Media Psychology. First, empirically, it shows that Weibo social-influence aggregation is organized into psychologically interpretable regimes (DN vs. SB) with large confirmatory contrasts on path length and modularity. These Cohen's *d* values (*d* = 2.71 for path length; *d* = 2.95 for modularity) are not merely “statistically significant confirmations” of a theoretical guess: they quantify a near non-overlapping separation of influence opportunity structures at the cascade level, establishing a measurable meso construct that laboratory attitude designs and emotion-coded virality studies have typically lacked. Second, methodologically, it offers an observational pipeline that separates regime definition from hypothesis testing and maps indicators onto process expectations (uncertainty reduction/social proof/brokerage), providing a meso layer for multi-level designs. Third, theoretically, it does not claim to have measured individual uncertainty, emotion, or personality; rather, it provides meso-level clues about how social influence is socially organized online and how those organizations can later be paired with measured psychological processes.

## 2 Literature review

This section situates the study in three strands: (i) classic and computational accounts of social influence and forwarding under uncertainty; (ii) cascade regimes as behavioral footprints of influence aggregation; and (iii) Chinese-platform cascade settings relevant to Frontiers Media Psychology. Table 1 summarizes prior emphases, remaining gaps, and the present design's responses.

**Table 1:** Summary of prior emphases, gaps, and the present study's response.

Prior emphasis	Remaining gap	Present response
Lab/attitude change under Weibo overload (Wang et al., 2021)	Limited meso account of natural cascade regimes	DN/SB regimes as observational aggregation signatures
Emotion-coded virality (Pivecka et al., 2022)	Needs full text; weak regime taxonomy	Structural regimes without emotion coding; future pairing
Collective anxiety/echo chambers (Yang et al., 2021; Song et al., 2024)	Community affect not cascade geometry	Modularity/path length as opportunity-structure indicators
Sentiment mitigation (ANSEC-MM/ECNS) (Rasheed et al., 2025; Ashraf, 2026)	Sentiment/veracity focus, not topology regimes	Topology-only DN/SB partition; no sentiment labels
True/false diffusion contrasts (Vosoughi et al., 2018)	Requires ground-truth labels absent in edgelists	Explicitly avoids veracity/AI labeling

### 2.1 Social influence, uncertainty, and forwarding in media psychology

Classic rumor psychology and contemporary computational social science converge on process expectations that motivate forwarding under incomplete information (Allport and Postman, 1947; Lazer et al., 2018). Uncertainty reduction leads users to seek confirmatory cues from peers and intermediaries; social proof increases the perceived appropriateness of sharing when others have already relayed content; and social influence amplifies the diagnosticity of highly visible accounts and dense local clusters (Centola, 2010; Kwak et al., 2010). Complex-contagion research further suggests that some behaviors require reinforcement from multiple contacts, which favors modular clusters and longer influence chains rather than single-exposure broadcast (Centola, 2010; Banerjee et al., 2013).

These micro expectations do not automatically specify cascade geometry. Under sequential peer reinforcement, cascades may elongate across many hops while remaining relatively narrow early on. Under hub-driven broadcast, cascades may expand rapidly at hop 1 and remain shallow thereafter (Goldenberg et al., 2001; Weng et al., 2013; González-Bailón et al., 2011). For Media Psychology, the analytic task is to treat those geometries as meso signatures of influence aggregation—measurable without claiming that any particular cascade is more “anxious,” “persuasive,” or “false.”

Frontiers studies illustrate complementary micro and content pathways into this problem space. Experimental overload designs show that exposure conditions can change attitudes on Weibo (Wang et al., 2021); emotion-coding designs show that valence and arousal predict sharing (Pivecka et al., 2022); community studies link collective anxiety to topic-based online groups (Yang et al., 2021); and personality-related work connects trait inference to echo-chamber homophily (Song et al., 2024). Sentiment-cascade mitigation frameworks such as ANSEC-MM and ECNS likewise emphasize influencer monitoring and structural thresholds, but they target negative-sentiment detection rather than DN/SB regime contrasts without veracity labels (Rasheed et al., 2025; Ashraf, 2026). The present study is positioned as a meso complement to these lines: it asks how aggregated forwarding is structurally organized when individual process measures and full-text emotion scores are not available.

### 2.2 Cascade regimes as behavioral footprints of social influence

On microblogging platforms, each directed repost edge records an interpersonal influence pathway: one user relays content originating through another's branch (Kwak et al., 2010; boyd et al., 2010). During public events, high-visibility and ordinary accounts jointly allocate attention, producing hybrid media gatekeeping that is psychologically consequential for what audiences see and believe (Chadwick, 2013; Starbird et al., 2018). To compare influence ecologies, established cascade indicators are used (Newman, 2010; Blondel et al., 2008; Weng et al., 2013): average path length (sequential influence opportunities), modularity (local reinforcement clusters), betweenness (brokered attention control), and hop-layer branching (continued influence opportunity). Density and clustering are retained as completeness checks.

These indicators are not substitutes for questionnaires. They are behavioral-aggregation footprints of social influence, used here as Media Psychology observables. Uncertainty reduction, social proof, social influence, and brokerage serve as interpretive perspectives for reading regime differences; confirmatory claims remain H1–H2. Psychological meanings beyond those structural contrasts require future multi-level measurement and are not treated as demonstrated processes.

Prior misinformation studies report that false items may diffuse farther or faster than truth (Vosoughi et al., 2018; Del Vicario et al., 2016; Shao et al., 2018), yet such claims depend on ground-truth labels unavailable in edgelist-only archives. True/false or AI/human labeling is therefore withheld; structural regimes are compared instead (Zhou and Zafarani, 2021; Lazer et al., 2018). Figure 2 schematizes DN vs. SB as an elongated multi-hop relay vs. early hub broadcast.

**Figure 2:**
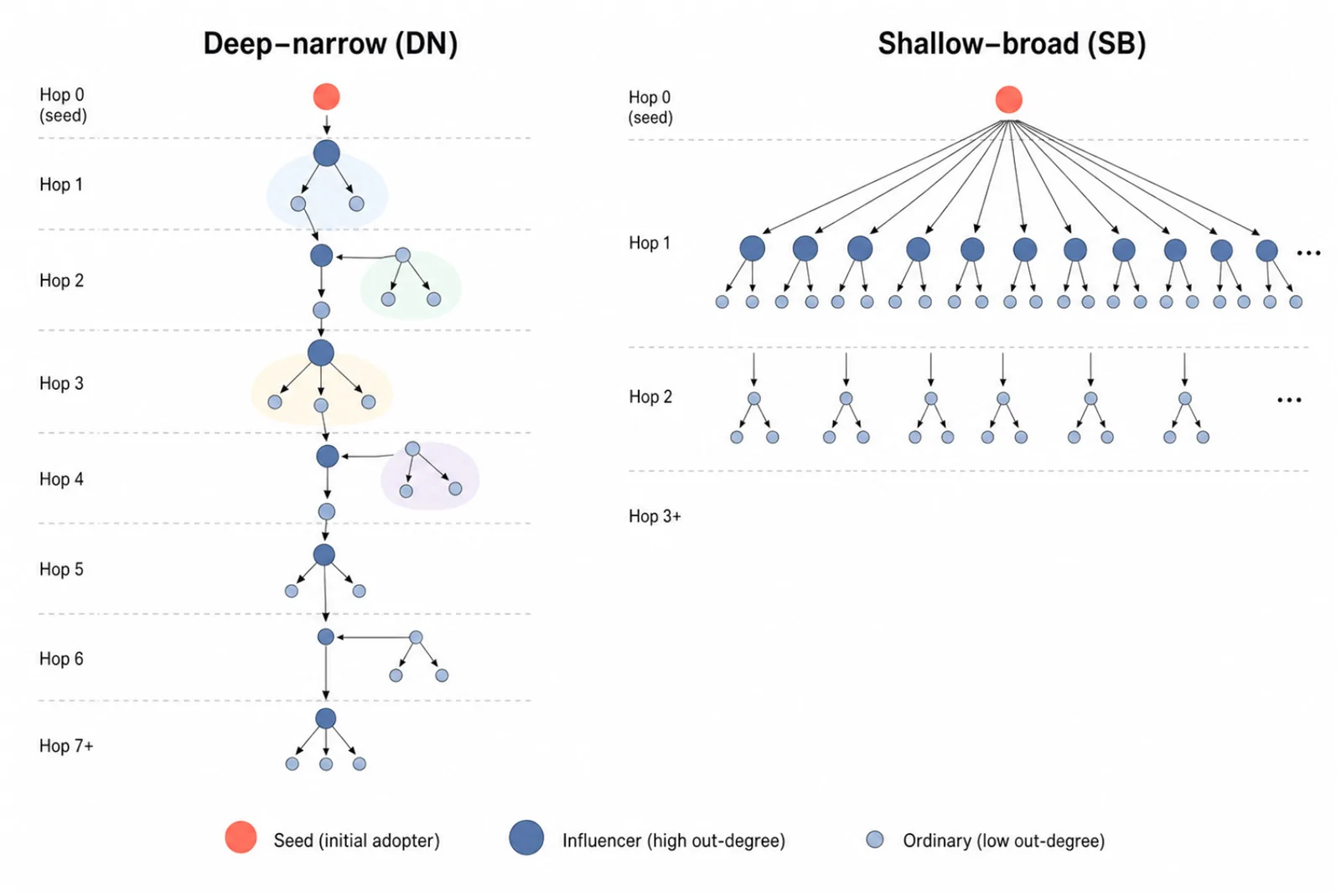
Conceptual schematic of deep–narrow (DN) vs. shallow–broad (SB) Weibo repost cascades. DN regimes elongate across many BFS hops with relatively constrained early breadth; SB regimes expand widely at early hops and remain shallower. Seed and branching-role nodes are illustrative only.

### 2.3 Chinese social media cascades and the present study setting

The Key Propagation Graph Generator (KPG)/The Chinese University of Hong Kong (CUHK) Weibo repost propagation corpus (November 2019–March 2022) provides recent Chinese-platform diffusion graphs with complete parent–child topologies (Zhang et al., 2025). Relative to early Twitter benchmarks, this setting is directly relevant to Frontiers media-psychology research already conducted on Weibo (Song et al., 2024; Wang et al., 2021; Yang et al., 2021). The corpus is treated as a public information-cascade context, explicitly not as a verified AI-generated misinformation corpus. Density and global clustering are retained as confirmatory completeness checks; sparse tree-like repost graphs often show near-zero clustering, and density or clustering are not hypothesized to separate regimes.

Taken together, the literature supports a Media Psychology niche: Weibo cascade regimes as meso indicators of social-influence aggregation, tested against process expectations (uncertainty reduction, social proof, brokerage) and kept distinct from unverified veracity or AI-content claims.

## 3 Materials and methods

This study examines social-influence aggregation as a Media Psychology phenomenon using observational records of public Weibo sharing. The pipeline reconstructs rooted repost cascades, classifies meso-level influence regimes, computes indicators mapped onto process expectations, and tests group differences with multiplicity control. Throughout, (a) features used to *define* regimes are separated from (b) indicators used to *test* hypotheses, so confirmatory claims are not circular.

### 3.1 Data source and ethical considerations

The KPG/CUHK Weibo propagation dataset is used (Zhang et al., 2025), a public corpus of repost trees collected between November 2019 and March 2022. In the Weibo22 release documented by the dataset authors, events comprise approximately 4,174 source posts and about 960,000 microblogs (reposts and comments) embedded in propagation graphs. Each line in the graph export encodes a repost node, its parent identifier, and a term frequency–inverse document frequency (TF–IDF) feature vector; directed trees are reconstructed exclusively from parent–child relations, and TF–IDF vectors are not used for clustering, hypothesis tests, or emotion scoring. The analytic unit is therefore a rooted cascade event rather than an ego network or an undirected friendship graph.

Critically for Media Psychology interpretation, the export used here does **not** include full microblog text. Message emotion (Pivecka et al., 2022) or individual attitudes (Wang et al., 2021) therefore cannot be scored. The study is an observational analysis of sharing-behavior aggregation—a legitimate Media Psychology design when framed as behavioral footprints of social influence—not a laboratory experiment or content-analytic emotion study. Aggregate event-level statistics only are reported; re-identification is not attempted. Because no participants were recruited and no instruments were administered, institutional human-subjects ethics review is not applicable; the design analyzes public behavioral traces.

#### 3.1.1 Inclusion, exclusion, and sampling flowchart

From the full tree catalog (*N*_trees_ = 4, 174): (i) 2,745 trees with fewer than 20 nodes and 415 trees with more than 250 nodes were excluded; (ii) 1,014 trees fell in the 20–250-node window, of which 1,013 had depth ≥1 and entered the eligible catalog; (iii) *k*-means assigned 399 DN and 614 SB events before sampling; (iv) balanced size-tertile draws retained 40 DN and 40 SB cascades (13/14/13 per tertile within each regime); (v) the analytic sample therefore retains 80/1, 013 ≈ 7.9% of eligible cascades. No additional content-based exclusions were applied. The 20–250 window was selected *a priori* to avoid near-degenerate tiny trees and mega-events whose BFS truncation would distort breadth/depth; it was not tuned *post hoc* to maximize DN/SB contrasts.

### 3.2 Cascade regime labeling and sampling

Regime labels are derived from cascade topology only. Over the eligible catalog, two shape descriptors are computed. Cascade depth *H* is the maximum BFS hop from the seed, indexing how far the longest relay chain extends. Cascade breadth *B* is the count of first-hop nodes (direct children of the seed). Relative breadth *B*_rel_ = *B*/*n*, where *n* is tree size, normalizes early broadcast by event size and thereby reduces the chance that large events are automatically labeled “broad” merely because they contain more nodes.

Both features are standardized to *z*-scores and clustered with *k*-means (*k* = 2). Implementation details: features are column-standardized; the production pipeline uses a deterministic two-means initialization at extremal depth vs. breadth points with up to 50 iterations to convergence; an independent sklearn KMeans check (*n*_init = 50, random_state=42, Lloyd algorithm) recovered an identical partition (adjusted Rand index = 1.0). Across 20 random seeds with *n*_init = 10, pairwise ARI among solutions was 1.0, indicating seed-stable recovery of the two-regime cut. The mean silhouette for *k* = 2 was 0.513, exceeding those for *k* = 3 (0.472), *k* = 4 (0.403), and *k* = 5 (0.387); thus, *k* = 2 is preferred on both the quantitative cluster-quality criterion and the theoretical DN/SB interpretation. Alternative *k*>2 solutions were inspected only as diagnostics and are not used for confirmatory tests; treating DN/SB as unique natural kinds is therefore not claimed. The cluster with higher mean depth is labeled deep–narrow (DN); the complementary cluster is labeled shallow–broad (SB). After clustering, balanced, size-tertile stratified samples (*n* = 40 per regime) are drawn with a fixed sampling seed (numpy Generator seed 42). Stratification partitions each cluster into size tertiles and samples evenly across tertiles, so that a single size band does not confound group contrasts.

Two interpretive constraints are binding. First, DN/SB labels are *not* ground-truth rumor categories, AI-generation indicators, or veracity scores (Vosoughi et al., 2018; Zhou and Zafarani, 2021). They are unsupervised topology regimes. Second, because depth and relative breadth define the clusters, they are reported only as descriptive characterizations of what the algorithm recovered. Treating depth as a confirmatory group outcome would be circular. Confirmatory inference is therefore reserved for density, average path length, clustering, and modularity—metrics that are mathematically related to, but not identical with, the clustering features.

#### 3.2.1 Structural dependence among metrics

In a rooted diffusion tree, average path length is structurally related to depth: longer longest-paths expand the set of attainable geodesics, so large DN–SB differences in *L*_*e*_ partly reflect geometry shared with the clustering feature *H*. Modularity can likewise be sensitive to elongated vs. star-like shapes because Louvain partitions respond to tree branching patterns. Accordingly, H1 is interpreted as a *characteristic* of the identified regimes—a theoretically predicted signature of chain-relayed opportunity structure—rather than as an entirely independent external validation of the DN/SB cut. Continuous OLS of *L*_*e*_ on depth and breadth (below) provides a complementary view that does not rely solely on the binary partition, while still acknowledging the structural depth–path relationship.

### 3.3 Node attributes and branching-role heuristics

For each node *i*∈*V*_*e*_ three attributes used in secondary analyses are recorded. Hop *h*(*i*) is the BFS distance from the seed and locates the node in the cascade's temporal–structural layering (under the assumption that hops approximate sequential relay order). Out-degree within *G*_*e*_ counts how many downstream reposts the node directly generates in the event graph. Branching-role proxy τ(*i*)∈{HB, MB, LB} (high-/medium-/low-branching) is assigned by within-cascade out-degree: HB if out-degree >8, MB if out-degree >3, else LB. Legacy manuscript wording “influencer/media/ordinary” referred to the same thresholds and is discontinued as occupational language; these categories are structural proxies only, not verified account identities. Sensitivity to nearby thresholds (>6/>2 and >10/>4) yields the same qualitative DN elevation pattern for high-branching brokerage in exploratory checks.

### 3.4 Indicators mapped to psychological process expectations

For each event graph *G*_*e*_ = (*V*_*e*_, *E*_*e*_) with *n*_*e*_ = |*V*_*e*_| and *m*_*e*_ = |*E*_*e*_|, four primary confirmatory metrics and several secondary descriptors are computed.

**Density** summarizes how completely nodes are interconnected (Equation 1):

$$\begin{array}{c} D_{e}=\frac{m_{e}}{n_{e}\left(n_{e}-1\right)}. \end{array}$$ (1)

In sparse repost trees, density is typically low; it is retained as a completeness check rather than as a hypothesized separator of regimes.

**Average shortest-path length** (over reachable ordered pairs) summarizes typical geodesic distance (Equation 2):

$$\begin{array}{c} L_{e}=\frac{1}{N_{\text{sp}}}\underset{i\neq j,d\left(i,j\right)<\infty }{\sum }d\left(i,j\right). \end{array}$$ (2)

Under the bridge model, longer *L*_*e*_ indexes chain-relayed influence *opportunity*—sites at which social proof or uncertainty reduction *could* operate—not evidence that those intrapersonal processes occurred (Allport and Postman, 1947; Centola, 2010). H1 predicts *L*_DN_>*L*_SB_.

**Global clustering** *C*_*e*_ measures local triadic closure (Newman, 2010). Directed trees reconstructed from parent–child reposts often contain few closed triangles, so *C*_*e*_ may approach zero even when substantive community structure exists at larger scales. Clustering is retained for confirmatory completeness but regime separation is not hypothesized.

**Modularity** *Q*_*e*_ is computed via Louvain community detection (Blondel et al., 2008) and quantifies the strength of community separation within the cascade subgraph. Higher modularity is consistent with fragmented clusters that *may facilitate* local reinforcement—a meso opportunity structure—without demonstrating that local social proof or affective reinforcement actually occurred (Song et al., 2024; Yang et al., 2021). H2 predicts *Q*_DN_>*Q*_SB_.

Cascade depth $H_{e}=\underset{i}{\max}h\left(i\right)$ and breadth *B*_*e*_ = |{*i*:*h*(*i*) = 1}| are descriptive shape metrics only. Betweenness centrality *g*(*i*) indexes structural brokerage (Freeman, 1977). For exploratory H3, mean betweenness is aggregated by branching-role proxy within each regime (descriptive summaries follow the analysis pipeline's top-betweenness aggregation by role).

Finally, hop-layer mean out-degree provides a structural process signature. For each hop *h*, the mean out-degree of nodes with *h*(*i*) = *h* is computed. Persistent mid-hop branching under DN would support the interpretation that chain-relayed regimes continue to generate opportunities for local influence after the initial broadcast window has passed. This quantity is explicitly *not* an emotion or anxiety trajectory (Pivecka et al., 2022; Yang et al., 2021).

### 3.5 Statistical analysis plan

Primary confirmatory metrics are *M*∈{*D, L, C, Q*}. For each metric *H*_0_:μ_DN_ = μ_SB_ is tested with Welch two-sample *t*-tests (α = 0.05, two-sided), which do not assume equal variances. Because four primary tests are conducted on the same sample partition, Holm–Bonferroni adjustment is applied and Holm-adjusted *p*-values are reported. Effect sizes are Cohen's *d* using the pooled standard deviation (Cohen, 1988). Conventions *d*≈0.2/0.5/0.8 are interpreted as small/medium/large, with the caveat that network metrics can yield larger standardized effects when regimes are structurally distinct.

As a non-circular complement to H1—and not as fully independent evidence free of structural dependence—a supplementary ordinary least-squares (OLS) regression of average path length on continuous depth and breadth is fit across all *N* = 80 events (Equation 3):

$$\begin{array}{c} L_{e}=\beta_{0}+\beta_{1}H_{e}+\beta_{2}B_{e}+\varepsilon_{e}. \end{array}$$ (3)

Predictors enter in their natural units (depth in hops; breadth as first-hop count). Reported quantities include coefficients, standard errors, 95% confidence intervals, *R*^2^, variance inflation factors (VIFs), and Cook's distance screening for influential points. Secondary analyses (hop-layer out-degree profiles; mean betweenness by branching role) are descriptive/exploratory and are *not* subjected to the same four-test Holm family.

Analyses used Python 3.12.10 with networkx 2.8.8 (shortest_path_length, density, average_clustering, betweenness_centrality; Louvain via community detection on the undirected projection of each cascade), scipy 1.15.2 (Welch tests), pandas 2.x/3.x, matplotlib 3.10, scikit-learn 1.9 (cluster diagnostics), and statsmodels 0.14 (OLS). Random seeds for *k*-means diagnostics and sampling are fixed as above.

### 3.6 Robustness and reproducibility safeguards

Three design choices guard against overclaiming. First, depth and relative breadth are withheld from confirmatory group tests. Second, Holm adjustment controls family-wise error among the four primary metrics. Third, size-tertile stratification reduces dependence on a single size band. Residual threats remain: alternative clustering features, different size windows, or different *k* could shift membership at the margin. DN/SB are therefore treated as analytically useful regimes rather than as unique natural kinds, and continuous OLS is reported alongside the discrete contrast. Results are reproducible from the public KPG Weibo graph files (Zhang et al., 2025); processed tables and code are available from the corresponding author upon reasonable request.

## 4 Results

### 4.1 Sample overview and descriptive regime recovery

*N* = 80 rooted repost cascades from the KPG/CUHK Weibo corpus were analyzed under the eligibility and sampling rules above (Table 2). The design yields a balanced comparison: 40 DN and 40 SB events. The merged union of sampled cascades comprises *N* = 6, 527 nodes and *M* = 6, 447 directed edges, indicating a sparse forest of trees rather than a densely interconnected social graph—as expected for event-wise repost cascades.

**Table 2:** Sample overview of KPG Weibo repost cascades by *k*-means topology regime.

Item	DN (deep–narrow)	SB (shallow–broad)
Number of cascade events	40	40
Pre-sampling cluster pools	399	614
Labeling basis	*k*-means on *z*(depth), *z*(relative breadth)	
Cluster quality (*k* = 2 silhouette)	0.513 (best among *k* = 2–5)	
Sampling	Balanced size-tertile draws within each cluster	
Tertile draws (S/M/L)	13/14/13	13/14/13
Merged repost union	6,527 nodes; 6,447 directed edges	
Subgraph size constraint	20–250 nodes per event	
Branching-role proxies	HB/MB/LB (out-degree >8 / >3 / else)	

Descriptively—and only as cluster characterization—mean depth was 5.20 for DN vs. 2.58 for SB (standardized contrast *d* = 1.99 if reported as a descriptive Welch statistic). Mean first-hop breadth was 49.4 vs. 62.3 (*d* = −0.29), a smaller and non-decisive descriptive difference after size normalization entered clustering via relative breadth. These numbers confirm that the unsupervised procedure recovered elongated vs. earlier-broad regimes in the intended direction. They are *not* confirmatory hypothesis tests: depth helped define the groups. The confirmatory question is whether regimes that differ in shape also differ in path length, modularity, and brokerage in ways predicted by the bridge model.

### 4.2 H1: average path length as a signature of chain-relayed influence opportunity

Table 3 reports event-level means and Welch contrasts for the primary metrics. Supporting H1, DN cascades showed substantially longer average path lengths than SB cascades (*M* = 3.235 vs. 2.218; *t* = 12.10, *p*_Holm_ < 0.001, Cohen's *d* = 2.71). The standardized effect is very large by conventional benchmarks, indicating that the geodesic structure of DN and SB events is not a subtle difference but a regime-level separation.

**Table 3:** Network metrics by cascade regime (DN vs. SB).

Metric	DN mean	SB mean	*t*	*p*	*d*	Sig.
Density	0.0177	0.0231	−1.78	0.1565	−0.40	
Avg path length	3.235	2.218	12.10	< 0.001	2.71	^***^
Clustering	0	0	—	1	—	
Modularity	0.551	0.200	13.21	< 0.001	2.95	^***^
Cascade depth^d^	5.20	2.58	8.88	—	1.99	—
Cascade breadth^d^	49.4	62.3	−1.31	0.195	−0.29	—

Primary *p*-values are Holm–Bonferroni adjusted; shape metrics are descriptive only. For primary metrics, *p* is Holm-adjusted. ^d^Descriptive shape metric (used in clustering; not confirmatory). $***p_{\text{Holm}}<0.001$.

Structurally, longer average path length means that—conditional on reachability within the event—information typically travels through more sequential relays under DN. Each additional hop is a potential decision point at which forwarding *could* be facilitated by uncertainty reduction, peer endorsement, or intermediary visibility (Allport and Postman, 1947; Centola, 2010; Kwak et al., 2010); the present data do not show that those psychological processes occurred. By contrast, shorter geodesics under SB are consistent with early hub saturation: many nodes are close to the seed or to early broadcasters, so the cascade's influence diameter remains compact. H1 therefore supports the meso claim that DN regimes instantiate chain-relayed social-influence *opportunity structures*, whereas SB regimes instantiate broadcast-window ecologies. Because *L*_*e*_ is mathematically related to depth in rooted trees, H1 characterizes the identified regimes rather than providing a fully independent external validation of the DN/SB cut.

The supplementary OLS model (*N* = 80) yields *R*^2^ = 0.756 (Adj. *R*^2^ = 0.749). Coefficients (SE; 95% CI) are: intercept 1.756 [0.091; (1.575, 1.938)]; depth β = 0.293 [0.019; (0.255, 0.332)], *p* < 0.001; breadth β = −0.00304 [0.00081; (−0.00465, −0.00143)], *p* < 0.001. VIFs for depth and breadth are both ≈1.01, indicating negligible multicollinearity. Shapiro–Wilk *p* = 0.115 for residuals does not indicate strong non-normality; Jarque–Bera *p* = 0.054. Three observations exceed the Cook's-distance rule of thumb 4/*n*, with maximum Cook's *D* = 0.286; refitting after winsorizing these points leaves the depth coefficient positive and significant. OLS therefore shows that continuous shape tracks path length smoothly, while still reflecting the structural depth–path relationship rather than a claim of causal independence from H1.

### 4.3 H2: modularity as a signature of fragmented local-reinforcement opportunity

Supporting H2, modularity was markedly higher under DN than under SB (*M* = 0.551 vs. 0.200; *t* = 13.21, *p*_Holm_ < 0.001, *d* = 2.95). Again, the effect size is very large. High modularity indicates that Louvain communities within DN cascades are more cleanly separated: within-module edge density is higher than in a random null, whereas SB cascades are less partitioned.

In media-psychology terms, modular fragmentation creates structural conditions that *may facilitate* local social proof inside clusters, while cross-cluster exposure may remain limited unless brokers intervene (Centola, 2010; Burt, 2004). This is compatible with—but not evidence for—echo-chamber homophily and localized affective climates discussed in Frontiers research (Song et al., 2024; Yang et al., 2021). Modularity is a graph property; personality homophily and collective anxiety are psychological constructs that would require additional measurement layers that are unavailable here. H2 establishes a meso-structural precondition (opportunity structure), not the micro content of modules.

### 4.4 Completeness checks: density and clustering

Density did not separate regimes after Holm adjustment (*M* = 0.0177 vs. 0.0231; *t* = −1.78, raw *p* = 0.078, *p*_Holm_ = 0.156, *d* = −0.40). Both regimes are sparse, as expected for tree-like repost graphs; SB events are descriptively slightly denser on average, but the difference is neither robust to multiplicity control nor large enough to carry the theoretical argument. Global clustering was effectively zero in both groups. In directed parent–child trees, closed triangles are rare because a child typically links to one parent rather than forming mutual triads. Clustering is therefore uninformative as a regime discriminator here, despite its importance in undirected social graphs (Newman, 2010). Retaining it in the confirmatory set documents that the DN/SB contrast is selective: path length and modularity move; density and clustering do not.

Figure 3 visualizes the primary metric distributions. Path length and modularity show clear regime separation with limited overlap, matching the large Cohen's *d* values. Density distributions overlap substantially; clustering is degenerate at zero. Figure 4 provides qualitative intuition using representative layouts: the DN exemplar elongates across many BFS layers with visible modular pockets, whereas the SB exemplar expands early and terminates sooner. These figures illustrate the statistics; they do not replace the confirmatory tests.

**Figure 3:**
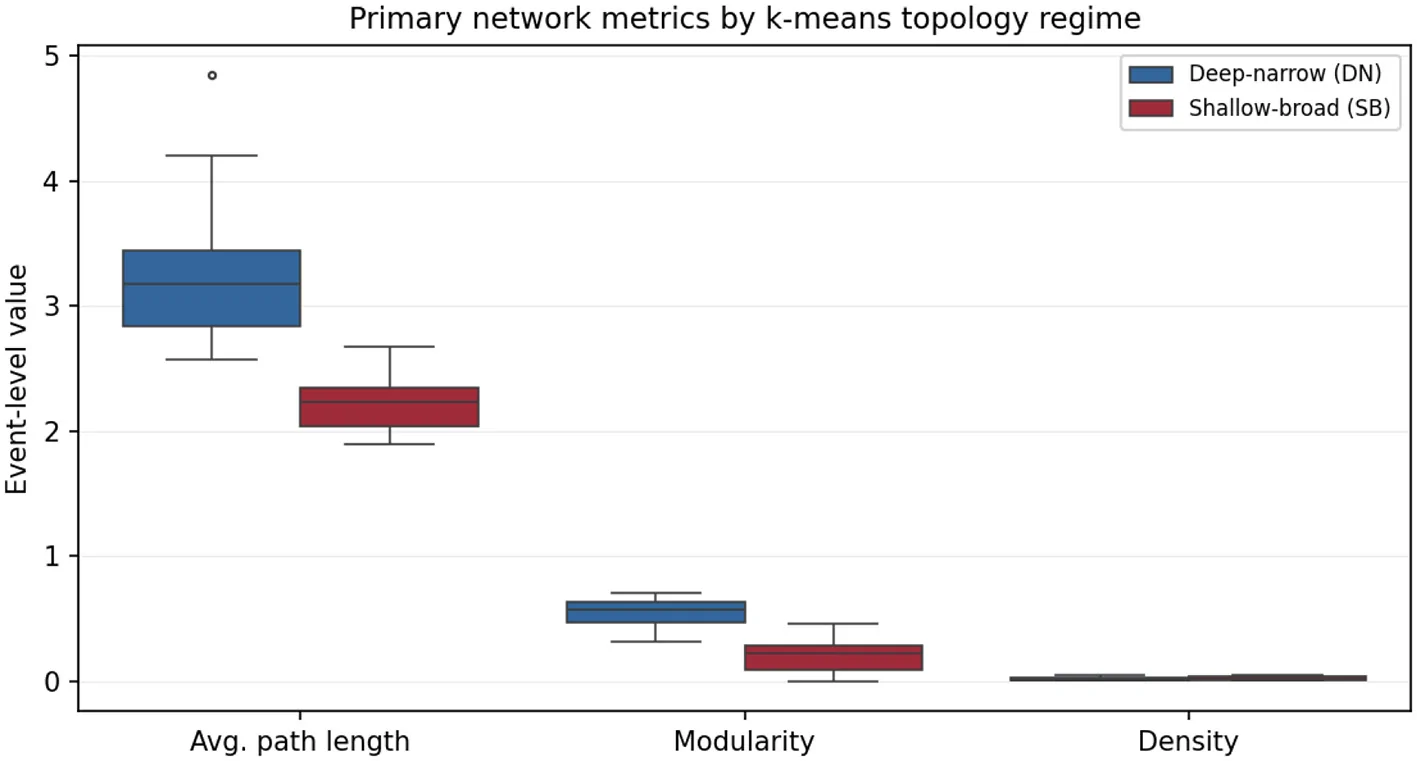
Distributions of primary network metrics by cascade regime (DN vs. SB; *N* = 80 events). Path length and modularity separate groups; density and clustering do not after Holm adjustment.

**Figure 4:**
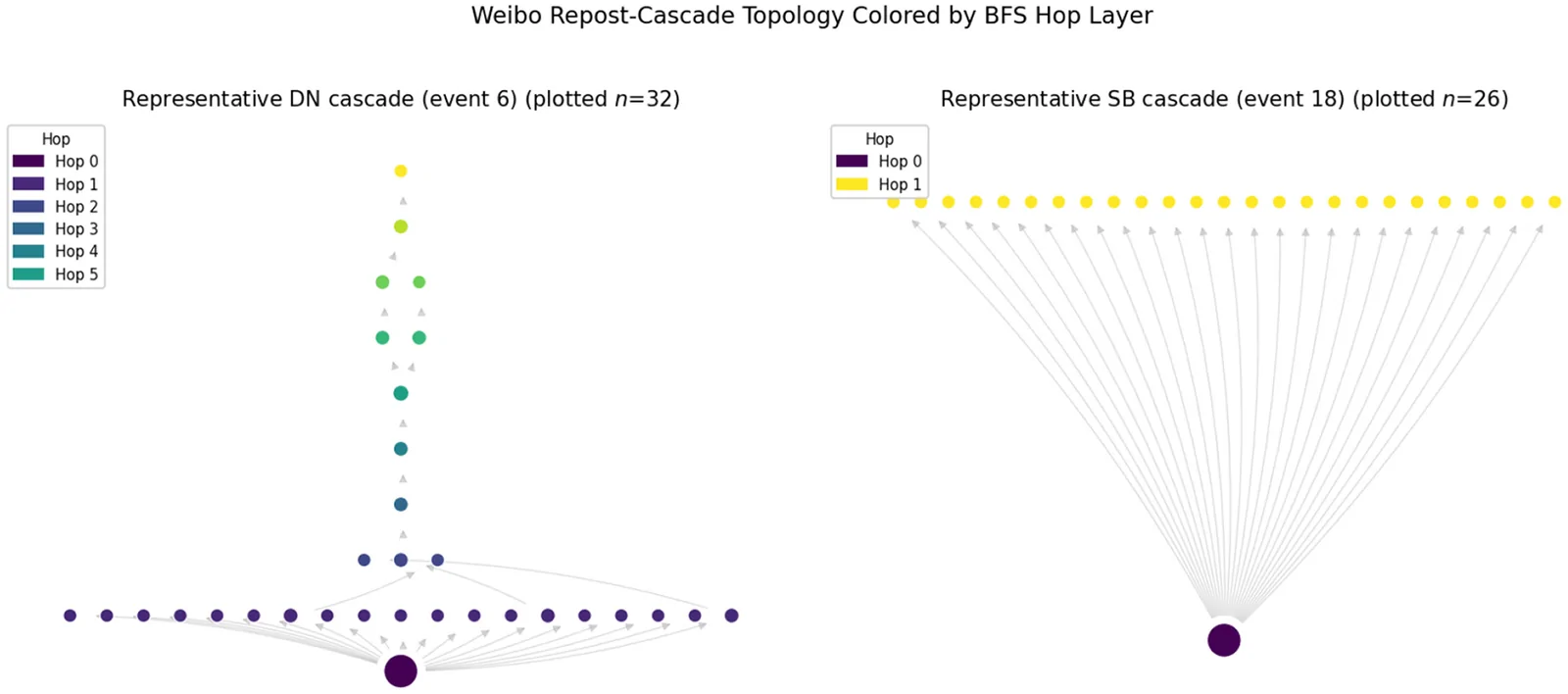
Representative DN vs. SB Weibo repost-cascade topologies colored by BFS hop layer. The DN exemplar shows an elongated multi-hop structure; the SB exemplar shows early breadth with limited depth.

### 4.5 Hop-layer branching: process evidence elaborating H1

If DN regimes reflect continued social-influence chaining, branching should persist at deeper hops rather than collapsing immediately after the first broadcast. Table 4 and Figure 5 summarize hop-resolved participation and mean out-degree (with standard deviations in the underlying tables).

**Table 4:** Hop-layer mean out-degree (and node counts) by cascade regime.

Hop	DN out-degree	DN nodes	SB out-degree	SB nodes
0	49.375	40	62.275	40
1	0.516	1,975	0.124	2,491
2	0.324	1,019	0.208	308
3	0.397	330	0.109	64
4	0.611	131	0.286	7
5	0.338	80	0.000	2

**Figure 5:**
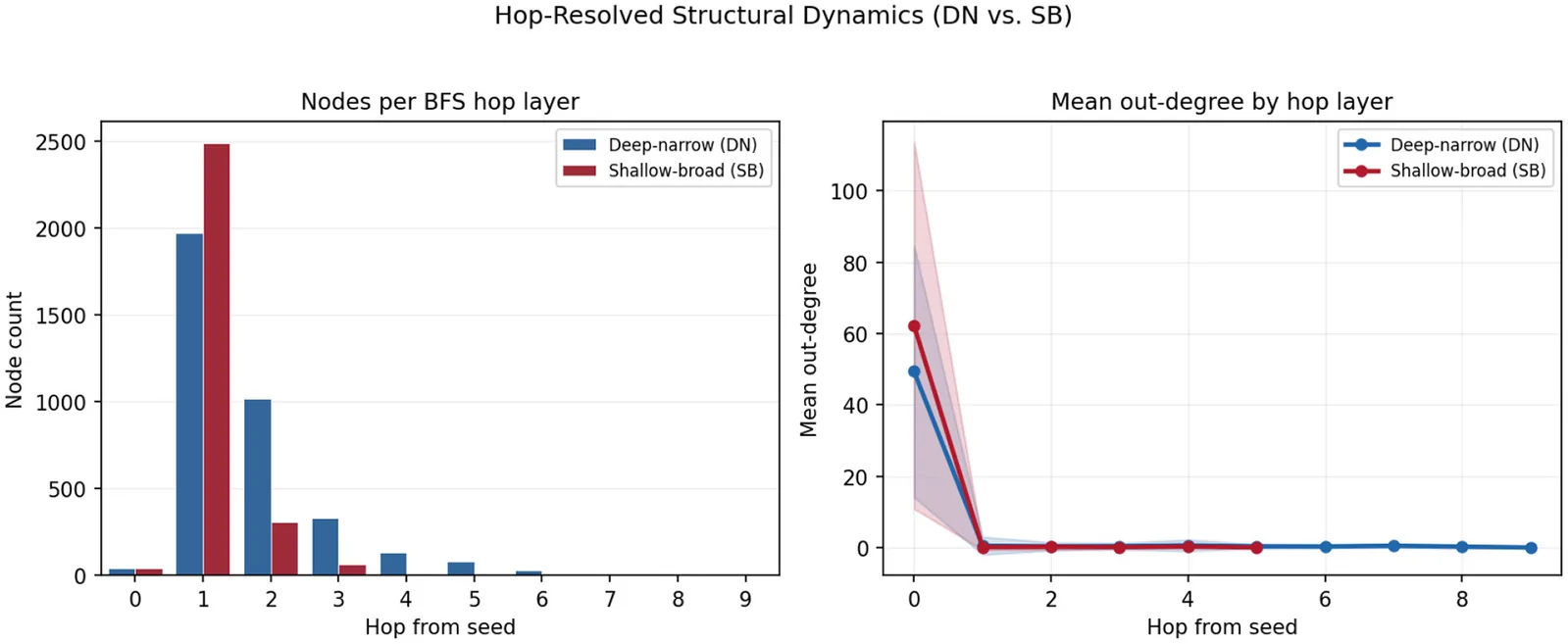
Hop-resolved structural dynamics for DN vs. SB cascades. Left: node participation by BFS hop. Right: mean out-degree (±1 SD) by hop. Curves index branching, not affect.

At hop 0 (seeds), SB events show higher mean out-degree (*M* = 62.3, SD = 51.4) than DN events (*M* = 49.4, SD = 35.2), consistent with broader early broadcast from the seed. At hop 1, however, the pattern reverses sharply: DN nodes retain higher mean out-degree (0.516 vs. 0.124), and DN still concentrates substantial participation (1,975 nodes) alongside SB's larger hop-1 mass (2,491 nodes). At hop 2, DN participation remains an order of magnitude larger than SB (1,019 vs. 308 nodes). Critically for the process reading of H1, at hop 3 DN mean out-degree remains elevated (0.397 vs. 0.109), with 330 DN nodes still active vs. only 64 SB nodes. By hop 4, SB participation has nearly collapsed (7 nodes), whereas DN still shows 131 nodes and a mean out-degree 0.611. DN events continue, with declining counts, through hops 5–9; SB events effectively end by hop 5.

Taken together, the hop profiles depict two diffusion rhythms. SB cascades front-load expansion at the seed and thin out quickly. DN cascades sustain mid-depth branching, creating repeated local opportunities for forwarding after the initial visibility window. This is the structural footprint expected if sequential social proof and intermediary relays matter more under DN than under SB (Romero et al., 2011; Weng et al., 2013); it does not demonstrate that those psychological processes occurred.

These curves must be interpreted with discipline. They describe layer-wise participation and local branching; they are *not* emotion, anxiety, or persuasion trajectories. They parallel—but do not replace—affective virality analyses that code message valence and arousal (Pivecka et al., 2022) or community-level affective climates (Yang et al., 2021). Their media-psychology value is operational: “continued influence opportunity” becomes a measurable structural quantity that future multi-level studies can align with coded content or self-reported motives at each hop.

### 4.6 Exploratory H3: brokerage across branching-role proxies

Table 5 reports mean betweenness by branching-role proxy and regime. Consistent with the exploratory pattern anticipated in H3, betweenness is descriptively elevated under DN for high-branching (0.003781 vs. 0.000216), medium-branching (0.003215 vs. 0.001377), and low-branching (0.004603 vs. 0.000722) proxies. Because H3 is outside the Holm confirmatory family, these contrasts are not claimed as confirmatory hypothesis support; “H3 is supported” language is therefore not used. The pattern is interpreted as a hypothesis-guided description of brokerage concentration under DN vs. SB.

**Table 5:** Mean betweenness centrality by branching-role proxy and cascade regime (exploratory; not Holm-adjusted).

Branching-role proxy	DN mean betweenness	SB mean betweenness
High-branching (HB)	0.003781	0.000216
Medium-branching (MB)	0.003215	0.001377
Low-branching (LB)	0.004603	0.000722

First, the DN elevation appears *across* role proxies rather than in a single elite category. That suggests regime structure—elongated modular trees—creates brokerage opportunities more than any one heuristic class monopolizes them. In DN events, even low-branching high-betweenness nodes can sit on paths that connect separated branches; in SB events, short geodesics and weaker modularity leave fewer such bridging positions.

Second, the combination of H2 (high modularity) and the exploratory H3 pattern describes a coherent gatekeeping ecology: modules are separated, yet some nodes occupy the structural holes between them (Burt, 2004; Chadwick, 2013). For influence interventions and corrections, this implies that messages seeded inside one module may fail to reach others unless routed through high-betweenness intermediaries (Starbird et al., 2018; Zheng et al., 2022). SB regimes, by contrast, resemble broadcast windows in which early hub visibility dominates and brokerage is less structurally decisive after the first hops.

Figure 6 recolors representative topologies by branching-role proxy; Figure 7 shows modular communities in the DN exemplar. These visualizations make the H2–H3 conjunction intuitive: DN layouts display separated color modules and dispersed bridging opportunities, whereas SB layouts are dominated by early expansion. Role labels remain degree heuristics within the event graph, not verified occupational identities.

**Figure 6:**
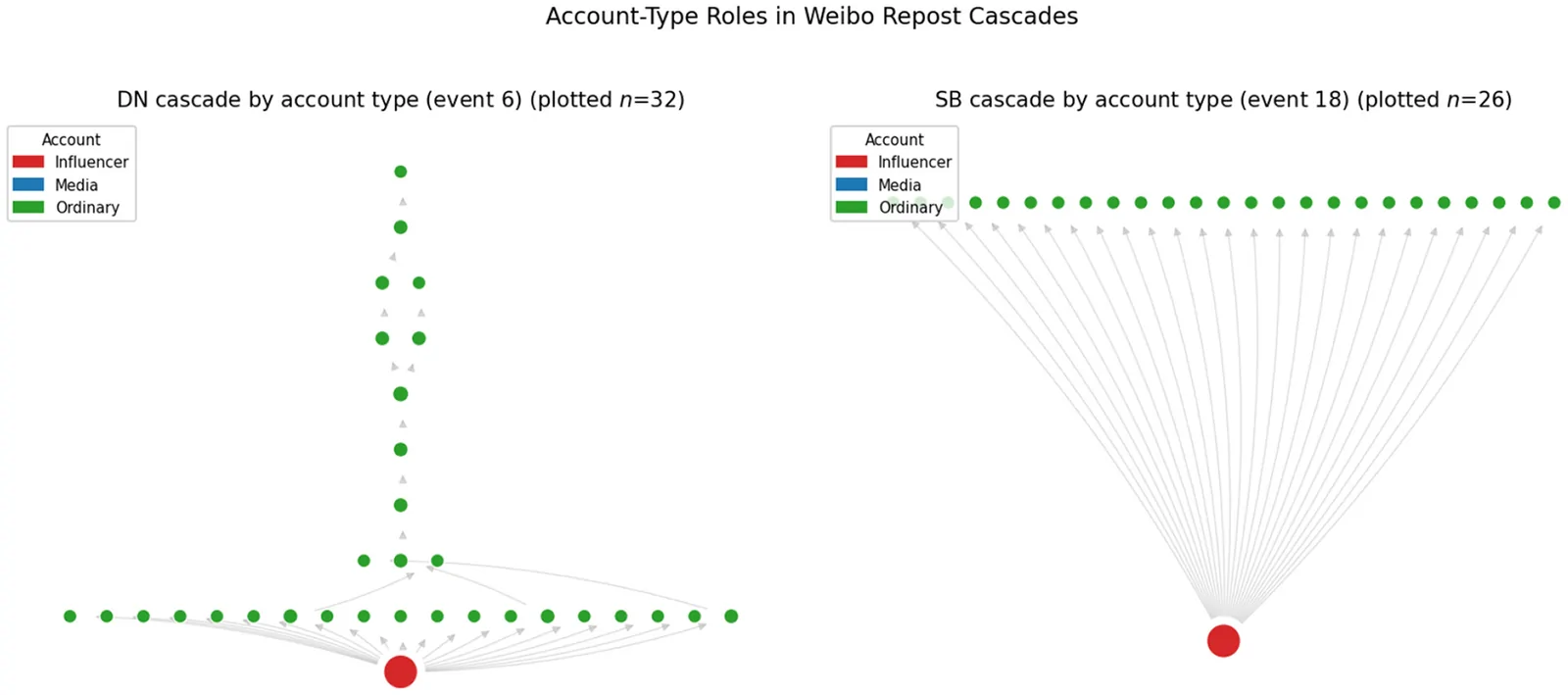
Representative DN and SB cascades colored by branching-role proxy (HB/MB/LB). Node size scales with degree; seeds are enlarged. Roles are structural proxies, not verified identities.

**Figure 7:**
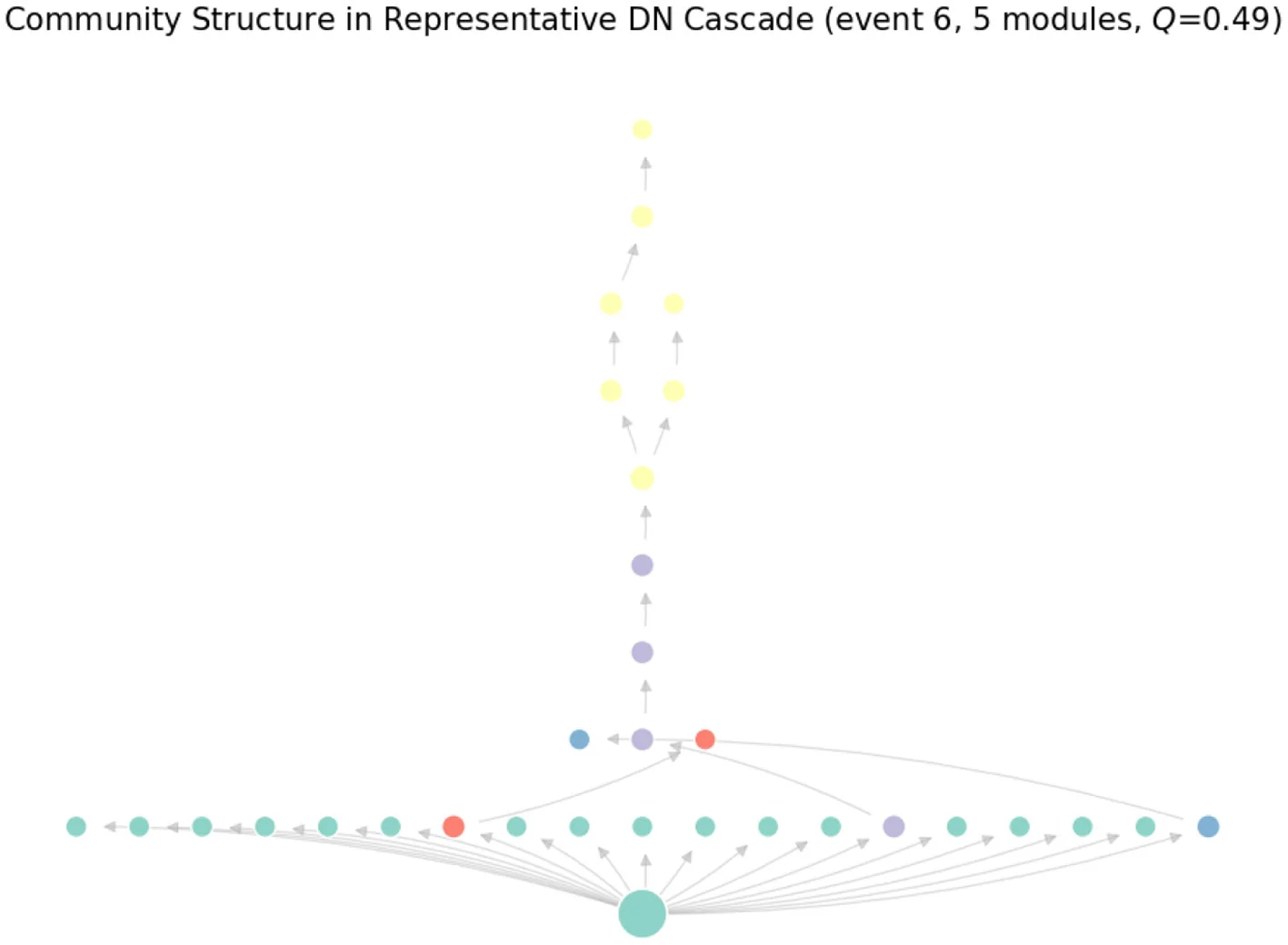
Modular community structure in the representative DN cascade. Colors denote Louvain modules; layout follows BFS hop layers. High modularity under DN (H2) is illustrated qualitatively here.

### 4.7 Integrated empirical summary

Across confirmatory and secondary analyses, social-influence aggregation on Weibo is regime-structured. DN regimes show longer paths (H1) and higher modularity (H2) after Holm correction; density and clustering do not discriminate. Hop-layer profiles show continued mid-depth influence opportunity under DN, elaborating H1. Brokerage is descriptively elevated under DN across branching-role proxies (exploratory H3). Continuous OLS (*R*^2^ = 0.756) shows that shape tracks path length beyond the binary partition, subject to the structural caveats above.

What remains unmeasured is equally important for Media Psychology scope. Individual uncertainty, motives, emotions, personality, or attitude changes are not observed. The empirical contribution is a meso-indicator layer that process measures can later be paired with.

### 4.8 Robustness summary

Primary inferences rely on Holm adjustment across four confirmatory metrics; H1 and H2 survive that correction with large effect sizes (*d* = 2.71 and *d* = 2.95). Depth is not re-tested as a confirmatory group contrast. Continuous OLS of path length on depth and breadth provides a non-circular complement to H1 while acknowledging structural dependence. Size-tertile stratification and the fixed eligible-size window (20–250 nodes) reduce, but do not eliminate, sampling sensitivity; alternative clustering features and different *k* remain limitations discussed below. Secondary hop and brokerage analyses are interpreted as elaborations of H1–H2 and as exploratory description for H3, not as an additional confirmatory family.

## 5 Discussion

This section interprets confirmatory regime contrasts (H1–H2), clarifies the exploratory status of brokerage patterns (H3), situates the contribution relative to network science and Frontiers Media Psychology benchmarks, and states observational limits and priority extensions.

### 5.1 Principal findings for media psychology

From a Media Psychology standpoint, the key finding is that Weibo users' sharing behavior aggregates into at least two social-influence ecologies. Deep–narrow regimes create longer chains of sequential influence opportunity and more modular local-reinforcement structure, with descriptively stronger brokerage across branching-role proxies. Shallow–broad regimes concentrate exposure earlier and offer fewer mid-depth reinforcement opportunities. These patterns describe different structural contexts of online influence—chain-relayed opportunity vs. hub-broadcast exposure (Allport and Postman, 1947; Centola, 2010; Kwak et al., 2010)—not demonstrated by intrapersonal mechanisms.

The claim is deliberately scoped. Meso organization of social influence is demonstrated using observational behavioral traces. Intrapersonal states that produced each repost are not claimed to have been measured. That distinction keeps the manuscript inside Media Psychology (social influence/media effects aggregation) without overstating evidence.

### 5.2 Why this belongs in media psychology rather than pure network science

Editors and readers may ask whether cascade metrics are “too structural” for Psychology. The answer is process-first. Path length is interpreted as sequential influence opportunity; modularity as local reinforcement clustering; betweenness as brokered attention control; hop branching as continued influence opportunity. These interpretations are anchored in rumor psychology, social contagion, and hybrid media influence (Allport and Postman, 1947; Centola, 2010; Chadwick, 2013), and they are aligned with Frontiers Media Psychology work on Weibo attitudes, emotion and sharing, collective anxiety, and echo chambers (Wang et al., 2021; Pivecka et al., 2022; Yang et al., 2021; Song et al., 2024). Graph formulas are tools; the scientific object is social-influence aggregation in a media environment.

### 5.3 Alignment with Frontiers media-psychology benchmarks

Where Wang et al. (2021) show that exposure conditions change attitudes, the present results show that natural diffusion can take structurally distinct forms that imply different influence ecologies. Where Pivecka et al. (2022) show that message emotion predicts sharing, the present results show that sharing aggregates into DN vs. SB regimes that future work can annotate with emotion codes. Where Yang et al. (2021) and Song et al. (2024) examine collective affect and personality-linked closure, high DN modularity and brokerage offer cascade-level counterparts. Zheng et al. (2022) examine seeding under network structure; elevated DN betweenness implies regime-sensitive influence interventions. Where ANSEC-MM and ECNS emphasize expression-capacity monitoring for negative-sentiment cascades (Rasheed et al., 2025; Ashraf, 2026), the present contribution is complementary: it isolates topology regimes without sentiment or veracity labels.

### 5.4 What supported hypotheses mean—And do not mean

Support for confirmatory H1–H2 means DN events afford more sequential influence opportunities and stronger modular local clustering. The exploratory H3 pattern is consistent with stronger brokerage under DN but is not claimed as confirmatory support. These results do *not* mean DN cascades are more emotional, more persuasive, more anxious, or less truthful (Wang et al., 2021; Pivecka et al., 2022; Vosoughi et al., 2018). Those claims require measures that are lacking. The Media Psychology contribution is a validated meso layer for multi-level designs.

### 5.5 Implications for influence interventions and media effects

Under DN, corrections may need cross-cluster seeding via brokers (Starbird et al., 2018; Zheng et al., 2022). Under SB, early hub visibility dominates. For Media Psychology applications—health communication, rumor clarification, platform literacy—message strategy should be regime-sensitive rather than one-size-fits-all.

### 5.6 Limitations and priority extensions

Scope boundaries remain. No individual attitude, emotion, personality, or uncertainty constructs are measured; full text is unavailable for emotion coding; clustering/sampling choices may matter; role labels are degree heuristics on 2019–2022 Weibo trees. The balanced 40/40 design cannot estimate the natural prevalence of DN vs. SB regimes on Weibo (pre-sampling pools were 399 vs. 614 among eligible trees). Observational associations between cascade topology and network indicators do not establish causal psychological mechanisms: differences could arise from seed-account properties, topic/content characteristics, posting time, audience size, platform recommendation, external events, temporal change across 2019–2022, or other unobserved confounders that cannot be controlled with the available edgelist archive. Priority extensions: emotion-coded hop dynamics (Pivecka et al., 2022); personality overlays (Song et al., 2024); experimental coupling of attitude change with regime exposure (Wang et al., 2021); and self-reports of uncertainty/social proof among cascade participants.

## 6 Conclusion

Media Psychology is concerned with how media environments organize social influence. On Weibo, deep–narrow cascades show longer path lengths (*d* = 2.71) and higher modularity (*d* = 2.95) than shallow–broad cascades after confirmatory Holm-adjusted tests (H1–H2). Exploratory brokerage patterns and persistent mid-depth branching further characterize DN regimes as chain-relayed opportunity structures, while SB regimes resemble hub-broadcast exposure. These patterns identify two influence ecologies using observational behavioral traces and supply a meso indicator layer for future multi-level Media Psychology designs. Individual affect, attitudes, and uncertainty reduction are not measured here; structural opportunity should not be equated with demonstrated psychological process.

## Data Availability

Publicly available datasets were analyzed in this study. The original contributions presented in the study are included in the article. The underlying KPG/CUHK Weibo propagation corpus is publicly available at https://github.com/kkkkk001/KPG/tree/main/data/Weibo. Processed data and analysis code are available from the corresponding author upon reasonable request. Further inquiries can be directed to the corresponding author.
